# Methods in a longitudinal cohort study of late reproductive age women: the Penn Ovarian Aging Study (POAS)

**DOI:** 10.1186/s40695-016-0014-2

**Published:** 2016-01-27

**Authors:** Ellen W. Freeman, Mary D. Sammel

**Affiliations:** 1 Department of Obstetrics/Gynecology and Department of Psychiatry, 3701 Market Street, Suite 820 (Mudd Suite), Philadelphia, PA 19104 USA; 2 Center for Clinical Epidemiology and Biostatistics, Perelman School of Medicine, University of Pennsylvania, U.S, Philadelphia, USA

**Keywords:** Menopause, Menopause transition, Menopausal symptoms, Reproductive hormones, Midlife cohort

## Abstract

**Background:**

This report describes the methods utilized in the Penn Ovarian Aging Study (POAS), which is a longitudinal cohort study of hormone dynamics and menopausal symptoms of women in the menopause transition.

**Methods/Design:**

The cohort is a community-based sample of generally healthy women enrolled in the late reproductive years. The study population is a stratified random sample of African-American and Caucasian women, identified by random digit dialing.

Of the 1427 women who were identified as potentially eligible, 578 women were eligible after full screening; 75 % of the eligible women enrolled in the study (436/578). At Period 14 (14 years after study enrollment), 67 % remained active and were fully evaluated (293/436). Attrition was non-differential with respect to the sample characteristics.

The aims of the project overall are to 1) identify within-woman trends of reproductive hormones (estradiol, follicle stimulating hormone, hormone, lutinizing hormone, inhibin B, dehydroepiandrosterone, testosterone, and anti-mullerian hormone), cofactors such as race, body mass index (BMI), age, physical and behavioral symptoms, and their predictions of menopausal symptoms, and patterns around the final menstrual period; 2) identify associations of hormone dynamics with physical and behavioral symptoms that occur with ovarian aging and identify racial differences in these factors; 3) identify associations of genetic polymorphisms with levels and longitudinal trends in menopausal symptoms. The cohort consists of 436 late reproductive-age women at enrollment, and now has 18 years of approximately annual follow-up assessments. Menopausal stage based on concurrent menstrual dates is identified at each follow-up period.

**Discussion:**

Studies of the cohort have shown that hot flashes can occur well before menopause and extend 10 or more years beyond menopause for sizeable numbers of women; provide evidence for new-onset depressed mood in the menopause transition and show that the final menstrual period is pivotal in the increases in depressive symptoms prior to menopause and decreases postmenopausal; suggest that poor sleep is common in the late reproductive years but increases in relation to the final menstrual period in only a small proportion of women; and show effects of obesity on reproductive hormones in the menopause transition. To date, more than 50 studies of the cohort are published in medical journals, demonstrating the relevance of these data to the clinical care of mid-life women.

**Electronic supplementary material:**

The online version of this article (doi:10.1186/s40695-016-0014-2) contains supplementary material, which is available to authorized users.

## Background

More than 80 % of U.S. women experience physical or behavioral symptoms around menopause, most commonly hot flashes and night sweats, depressive symptoms, and sleep disturbances [1]. Although the severity of these symptoms varies widely, many women seek medical relief for distressing symptoms that disrupt their functioning. However, whether these symptoms are associated with the biological changes of ovarian senescence or with age-related changes and other behavioral and psychosocial conditions of mid-life has only recently been a target of scientific investigation, and knowledge of the efficacy of therapeutic treatments is limited.

Calls for research have aimed to increase understanding of biological and behavioral changes associated with ovarian aging, and to identify whether there are racial differences in these changes. Recent cohort studies of mid-life or late reproductive age women are elucidating associations between symptoms and reproductive aging and providing new information that can lead to better preventive and therapeutic strategies and reduce the short- and long-term morbidity of women’s mid-life and postmenopausal years.

The purpose of this report is to describe the methods in the Epidemiologic Study of the Late Reproductive Years, which is also termed the Penn Ovarian Aging Study (POAS), supported by the National Institute of Aging (RO1-AG12745). The POAS cohort consists of healthy, mid-life women in their late reproductive years, who were randomly identified through random digit dialing in Philadelphia County, Pennsylvania, with stratification to obtain equal numbers of African American and Caucasian women. The cohort has continued for 18 years with approximately annual follow-up evaluations.

POAS studies focus on hormone dynamics and associated symptoms of menopause. The participants were premenopausal at enrollment and then entered and moved through stages of the menopause transition. The overall aims of the project are to 1) identify how within-woman trends of reproductive hormones (estradiol, follicle stimulating hormone, luteinizing hormone, inhibin B, dehydroepiandrosterone, testosterone, and anti-mullerian hormone) and cofactors such as race, body mass index (BMI), age, physical and behavioral symptoms predict the progression through the menopause transition; 2) identify associations of hormone dynamics with physical and behavioral symptoms that occur with ovarian aging and identify racial differences in these factors; 3) compare hormone levels and longitudinal trends between African American and Caucasian women; 4) identify associations of genetic polymorphisms with levels and longitudinal trends and with menopausal symptoms.

## Methods

### Overview of study design

This longitudinal study has 14 complete assessment periods followed by 4 partial assessment periods for a total of 18 assessment periods. In each full assessment period, data were collected at 2 visits scheduled between days 2 and 6 of 2 consecutive menstrual cycles (or 1 month apart in non-cycling women). In the first five years of the project (Phase 1), the follow-up assessment periods were approximately 9 months apart. In Phase 2 (years 6–10) and Phase 3 (years 11–15), follow-up assessments were conducted annually. There were 14 complete assessment periods due to a one-year delay in funding in year 11. Limited follow-up was conducted annually by telephone interview for follow-up periods 15–17. Full follow-up with home visits was resumed in year 18.

Study visits were conducted at participants’ homes. At each visit, a trained research interviewer administered a structured interview questionnaire and obtained anthropometric measures and blood samples for the hormone assays. Participants also completed standard self-report questionnaires to assess physical, behavioral and mood factors and completed a daily symptom report for 1 menstrual cycle at each assessment period.

### Sample selection and recruitment

A sample of 436 healthy women (218 African American, 218 Caucasian) was recruited in Philadelphia County over a 16-month period in 1996–1997. Recruitment was stratified by race to obtain equal numbers of African American and Caucasian participants. In a two-phase recruitment process, potentially eligible women were first identified through random-digit dialing, using a modified Mitoksky-Waksberg method [2]. The women who were identified through random digit dialing were then contacted by a research interviewer, who explained the details of the study and screened for study eligibility.

Women were eligible for study participation if they were between 35 and 47 years of age, experienced regular menstrual cycles in normal range (22–35 days) in the past 3 months, and had at least one ovary and a uterus. Women were excluded from enrollment if they were pregnant, taking hormone therapy or using hormonal contraception, taking psychotropic medications, had a history of illness that could affect hormonal function (e.g., diabetes, liver disease, breast or endometrial cancer, et al.), had a history of drug or alcohol abuse or a major psychiatric disorder in the previous 12 months, or were non-English speaking.

The age range was carefully considered when the cohort was recruited in order to evaluate ovarian aging *prior to observable menstrual cycle changes.* The lower age limit of 35 years was selected as an age when follicular depletion accelerates, resulting in subtle increases in follicle-stimulating hormone (FSH) and decreases in inhibin [3], and is consistent with standard age groupings as in U.S. census data (e.g., ages 35–39 years). The upper age limit of 47 years was selected as an accepted median age for women entering the perimenopause, as shown in the estimates of Treloar [4] and McKinlay [5]. This provided a unique baseline of premenopausal women for the subsequent follow-up through the menopause transition.

A cohort size of *N* = 300 women (150 in each racial group) was predetermined using a 2-sided alpha error of 0.05 and 80 % power to detect clinically relevant differences in hormones and symptoms. The enrollment number was increased to 436 women to account for estimated dropout in the first 4 years of the study. However, it is noteworthy that retention far exceeded the initial estimate (see below), and the powered estimate of 300 participants remained at Year 12. Further post-hoc calculations of statistical power for hypothesis testing have remained strong throughout the years of the study.

To obtain the cohort of 436 women, 1427 potentially eligible women were identified through random digit dialing. Of these, 129 (9 %) could not be contacted further by the research staff, 308 (22 %) declined to participate without providing eligibility information, and 412 (29 %) were ineligible. Reasons for ineligibility included hysterectomy (*n* = 111), use of hormonal contraception (*n* = 87), menstrual irregularities or no menses (*n* = 92), known to be leaving the area during the study period (*n* = 31), medical contraindications (*n* = 30), pregnant, breast feeding or attempting pregnancy (*n* = 24), alcohol or drug abuse (*n* = 6). Of the 578 women eligible after full screen, 436 (75 %) enrolled, and 142 (25 %) declined. Neither eligibility nor participation differed significantly by race.

Informed consent for study participation and follow-up contact was approved by the Institutional Review Board of the University of Pennsylvania, signed and witnessed at the first study visit and repeated thereafter when there were protocol changes (e.g., the addition of another hormone assay). Verbal consent was given for telephone contacts in the screen period and at later follow-up telephone contacts.

### Sample characteristics at enrollment

Four- hundred-thirty- six women enrolled in the cohort (218 African American, 218 Caucasian). All participants were premenopausal with regular menstrual cycles in normal range (22–35 days) at enrollment. The mean age was 41.4 (SD 3.5) years (range 35–47 years). The mean cycle day of the blood draw was 4.0 (SD 1.0). Mean (SD) hormone levels were FSH: 8.3 (5.2) mIu/mL; inhibin b: 78.0 (77.0) pg/mL; LH: 3.4 (2.6) mIU/mL; estradiol: 44.1 (38.8) pg/mL. [6]. The mean BMI was 29.1; 38 % (166/436) were current smokers; 81 % were employed; 33 % were high school graduates, 11 % had less than high school education, 56 % had college or technical training beyond high school; 57 % were married, 43 % were single, divorced, separated or widowed.

### Data collection

a. *Interview questionnaire*. A structured interview questionnaire was constructed and tested for the study and administered by a trained research interviewer at the first visit of every assessment period. Primary components of the interview included standard demographic information; menstrual cycle characteristics (most importantly, the dates of the current and previous two menstrual periods); menopausal symptoms (using a validated menstrual symptom questionnaire [7], which were reported for the past month and the past year and included frequency and severity of each symptom; general and gynecological health (current, history and contraceptive use), all current medications; health practices and behaviors (including smoking and alcohol consumption); physical activity (adapted from the validated College Alumni Health Study questionnaire [8] with additional questions such as number of city blocks walked daily, etc., that are shown to be a major source of physical activity among African American women [9]; sleep disturbance (using the validated St Mary’s Hospital Sleep Questionnaire [10, 11]; decreased functioning due to symptoms (using the Sheehan Disability Scale [12]).

b. *The Menopausal Symptom List (MSL)* [7]. This validated symptom list included 12 common menopausal symptoms: hot flashes/night sweats; aches/joint pain/stiffness; depressed mood; poor sleep; decreased libido; vaginal dryness; urine leaks; headache; irritability; mood swings; anxiety; and concentration difficulties. The symptom list was embedded in the structured Interview Questionnaire and administered by the research interviewer at each assessment period (periods 1–18). For each symptom, the interviewer asked whether the symptom occurred in the past month, whether the symptom occurred in the past year, and asked the participant to rate the frequency (number per day, week or month) and severity (none, mild, moderate, severe) of each symptom. The MSL was validated for the study [7], and several reports of associations between the symptoms and menopausal stages are published [13, 14].

c. *Mood and anxiety diagnosis*. Current mood disorders were screened for all women at enrollment using the Symptom-Driven Diagnostic System (SDDS-PC) [15], which was developed from the Structured Clinical Interview for DSM III R Diagnosis (SCID) [16]. The SDDS is a validated screening measure for DSM diagnoses of major mood disorders, its symptom checklist is rapid and easily used by the participants, it provides symptom information collected systematically from all participants, and the screening information can be obtained in the telephone interview followed by in-person evaluation of screen positives at the next study visit.

Clinical diagnosis was made using the Primary Care Evaluation of Mental Disorders (PRIME-MD**)** [17] to obtain DSM diagnoses of depression and anxiety disorders at each assessment period through Period 6. The Prime-MD is a 2-stage system where the participant first completes a 26-item self-administered questionnaire that screens for 5 of the most common groups of disorders in primary care: depressive, anxiety, alcohol, somatoform and eating disorders. Algorithms are provided, and when the checklist scores indicate a defined severity level, trained interviewers administer a brief set of questions to determine a DSM diagnosis. The Prime-MD is validated and highly correlated with full SCID interviews for DSM diagnosis.

The Patient Health Questionnaire (PHQ) [18] was administered in Periods 7 through 14. The PHQ is a self-administered version of the Prime-MD to provide DSM diagnosis of major depressive disorder, other depressive syndromes, panic disorder, other anxiety syndromes, eating disorder and alcohol abuse. It also queries bothersome problems, current medications and reproductive events. The PHQ is extensively validated and has a sensitivity of 0.88 and specificity of 0.88 for the diagnosis of MDD [19].

d. *Behavioral symptom measures*. Six self-report questionnaires were completed by the participants at each assessment period. All measures are validated and published in the literature.

Depressive symptoms were evaluated at each assessment period using the Center for Epidemiologic Studies Depression Scale (CES-D) [20]. The CES-D is a 20-item self-report inventory that was developed for epidemiologic research, is widely used and validated. The standard CES-D cutoff score of 16 or greater indicates high depressive symptoms. A higher CES-D score of 25 or greater can be examined as a closer approximation of a clinical diagnosis of depression [17, 21].

Anxiety symptoms were evaluated at every assessment period using the Zung Anxiety Index, a validated, 20-item self-report measure that is sensitive to the frequency of both affective and somatic symptoms of anxiety [22]. Zung established score ranges to classify normal anxiety [20–35], mild to moderate anxiety [36–47], and high anxiety (48–60).

Perceived stress was evaluated using the Perceived Stress Scale (PSS) [23] at each assessment period. The PSS is a 14-item widely validated self-report measure of the degree to which situations are appraised as stressful.

Quality of life was evaluated with the Quality of Life Enjoyment and Satisfaction Questionnaire (Q-les-Q) [24], which assesses various aspects of quality of life. The 16-item summary scale was used in this project. The Q-les-Q has high reliability and validity, addresses general functioning rather than disease-specific issues, and has considerable normative data available. The Utian Quality of Life Scale (UQOL) [25] was added in Periods 11–14. This validated instrument is specific to a perimenopausal population and quantifies “sense of well-being”.

General health**.** The SF-12 Health Survey [26] was administered at each assessment period to monitor health outcomes. The SF-12 is a 12-item short form developed from the SF-36 Health Survey. There are two summary scales, which have demonstrated equivalence to the SF-36 and yield reliable summary scales for physical and mental health.

e. *Sleep disturbance*. The St. Mary’s Hospital Sleep Questionnaire (SMHSQ) [11], a standard, self-administered questionnaire, was administered to assess sleep disturbances at each assessment period through Period 8. We adapted the SMHSQ by adding items to assess the etiology of nocturnal awakenings, the frequency of sleep medication use, and whether the previous night of sleep was comparable to usual sleep and deleting items about bedtime, fall-asleep time, wake time, time out of bed, and the “depth” of sleep, resulting in a total of 20 items.

We subsequently conducted a factor analysis of the SMHSQ, using all data from the first assessment period [27]. The factor analysis identified 3 factors: sleep quality, sleep complaints, and sleep latency. The sleep quality factor explained the largest proportion of variability in responses (37 %) and was used as a primary outcome variable for sleep studies in the cohort [27, 28].

“Poor sleep**”** (frequency and severity) was included as a symptom in the validated menopausal symptom questionnaire, administered at each assessment period through 18 years of follow-up [7]. This sleep item had a high correlation with the sleep quality factor score derived from the SMHSQ (r = 0.83) [27] and has been used as a primary outcome measure in sleep studies in this cohort [28, 29].

The Women’s Health Initiative Insomnia Rating Scale (WHIIRS) [30] was collected in assessment periods 10–14. The WHIIRS is a 5-item self-report questionnaire that is sensitive to sleep disturbances over time and validated in a perimenopausal population. The Multivariable Apnea Prediction Index (MAP) [31] was administered in Periods 10–14).

f. *The Female Sexual Functioning Index (FSFI)* [32] was collected in each assessment period 8–14. The FSFI is a 19-item, multi-dimensional, self-report instrument for assessing the key dimensions of sexual function in women. Domains include desire, arousal, lubrication, orgasm, satisfaction, and pain. The instrument has been validated and scaled on a sample of women with clinically diagnosed female sexual dysfunction.

Symptoms of decreased libido and vaginal dryness were included in the validated Menopausal Symptom Questionnaire, administered by interview at each assessment period. The items were “Please tell me if you have experienced a decreased libido or interest in sex in the past month” and “Have you experienced vaginal dryness or discomfort in the past month.”

A 30-day and a 1-year time period were queried; participants rated the severity (none, mild, moderate, severe) and the frequency of the items.

g. *The Social Adjustment Scale (SAS)* [33] was completed by participants at assessment Periods 1–3, 6–8, and 11–13. The scale provides domain scores that evaluate the participant’s role performance, interpersonal relationships, friction, feelings and satisfaction in work, and social and leisure activities

h. *Cognition.* Brief tests of cognitive memory were completed at each assessment period. These included the Buschke-Fuld Selective Reminding Test [34], which provided scores for total recall, short-term recall and long-term storage; the Digit Symbol Substitution test (Weschler Adult Intelligence Test-III) [35], a sensitive measure of cognitive processing speed [36]; and the Digit Symbol Copy test (WAIS-III) [35] to assess sensorimotor processing speed. These are widely used, brief cognitive tests, with extensive data published in the literature.

i. *Daily Symptom Reports (DSR)* were completed by participants for one menstrual cycle (or one month if not cycling) at each assessment period to determine prospectively the occurrence and severity of symptoms in relation to the menstrual cycle. The DSR lists 20 symptoms specific to reproductive aging as described in the menopause literature. Participants rated the symptoms daily on a 5-point scale of severity according to written descriptors (0 = not present to 4 = very severe). The symptoms included on the DSR were also included in the Menopausal Symptom List (above), which was administered by interview at each assessment period.

j. *Anthropometric measures*. Measures of height, weight, waist and hip circumferences were made at each assessment period 1–14. Height (without shoes) was measured to the nearest 0.5 cm with a vertical ruler. Body weight (light clothing only) was measure to the nearest 0.2 kg. Waist circumference was measured at the maximum abdominal girth, in duplicate, to the nearest 0.5 cm. Hip circumference was measured at the maximal protrusion of the hips at the level of the symphysis pubis, in duplicate, to the nearest 0.5 cm. The average of duplicate measures was calculated for analysis.

Body mass index (BMI) was calculated by computer algorithm from the measures of height and weight at each assessment period, using the average of the duplicate measures of height and weight at each assessment period (weight (kg) divided by the square of height (cm).

k. *Hormone measures*. Blood samples were obtained at each study visit (2 visits per assessment period). Visits were scheduled throughout the day in days 2–6 of the menstrual cycle or approximately one month apart in the assessment period for non-cycling women. Standardizing the collection of blood samples to the first 6 days of the menstrual cycle was maintained for cycling women throughout the project*.* Less than 1 % of the blood samples from cycling women were outside the 6-day window.

Blood samples (2 ½ ounces, non-fasting) were drawn from the non-dominant arm into vacutainer serum separator (tiger top) tubes containing separator gel and clot activator. The tubes were kept on ice, centrifuged for 20 min and frozen in aliquots (−80 C.) using polypropylene tubes.

Hormone values were measured by radioimmunoassay in the Clinical and Translational Research Center (CTRC) of the University of Pennsylvania. Assays were conducted in batched samples of 20 participants, with 4 samples at 4 time points per participant, to reduce the within-subject variability due to assay conditions. Estradiol, follicle stimulating hormone (FSH), luteinizing hormone (LH), dehydroepiandrosterone (DHEAS), testosterone, and sex hormone binding globulin (SHBG) were assayed using commercial kits (Coat-a-Count, Diagnostic Products Corp). Anti-mullerian hormone (AMH) assays were conducted using ELISA commercial kits (GEN 2, Beckman Coulter). Assays of Inhibin b were initially conducted in the laboratory of Dr. Patrick Sluss (Massachusetts General Hospital, Boston, MA) (assessment periods 1–10), and then conducted in the CTRC (assessment periods 11–14) using a commercial kit (Diagnostic Systems). Assays were performed in duplicate for all hormones and repeated if values differed by more than 15 %. Inter- and intra-assay coefficients of variation were calculated for the study samples in each study phase and were consistently less than 5 %.

l. *Genomic DNA and genetic polymorphisms*. Genomic DNA was obtained from 95 % of the cohort (413/436). Extraction of genomic DNA was performed using the QIAamp 96 DNA Buccal Swab Biorobot Kit and performed on a 9604 Biorobot (Qiagen, Inc., Valencia, CA). We identified seven genes involved in the downstream metabolism of estrogen and chose functionally relevant SNPs with a sufficiently high allele frequency to provide adequate power for testing first order interactions in the cohort. These SNPs were: *COMT* Val158Met (rs4680), *CYP19* Arg 264Cys, (re700519), CYP1A2*1 F (rs762551), CYP1B1*4 (Asn452Ser, rs1800440), CYP1B1*3 (Leu432Val, rs1056836), CYP3A4*1B (rs2740574), * SULT1A1* Arg213His (*2; rs9282861), SULT1A1*3 (Met223Val, rs1801030), *SULT1E1 (−*64G > A Promoter Variant; rs3736599), and *SULT1E1* A220G 3’UTR Variant (rs3786599). Genotypes were determined using previously described methods [37, 38].

m. *Other behavioral measures*. Additional validated behavioral measures were included at selected assessment periods to assess variables for pre-specified studies. The validated Kaiser Physical Activity Survey (KPAS) [39] was included in Periods 13–14. The Bristol Female Lower Urinary Tract Symptoms Scored Form (BFLUTS-SF) [40] was administered at Periods 11–14. A 61-item Dietary Assessment Questionnaire (DAQ) from the Nurses’ Health Study [41] was completed by participants at Periods 2, 5, 6, 7, and 9. Table 1 presents a summary of the outcome measures by study visits.

### Definition of menopausal status

We defined 5 stages of the menopause transition based on menstrual bleeding patterns and adapted from the initial Stages of Reproductive Aging Workshop (STRAW) [42] in order to capture the early changes in the menopausal transition [43]. The following 5 categories were defined in the cohort: 1) Premenopausal: regular menstrual cycles in the 22–35 day range. (Note: all participants were premenopausal at cohort enrollment). 2) Late premenopausal: a change in cycle length of 7 days or more in either direction from the participant’s personal baseline at enrollment in the cohort and observed for at least one cycle in the study. 3). Early transition: changes in cycle length of 7 days or more in either direction from the participant’s personal baseline at enrollment in the cohort and observed for at least 2 consecutive cycles in the study or 60 days amenorrhea. 4). Late transition: more than 60 days to 11 months amenorrhea. 5). Postmenopausal: 12 months or more amenorrhea, excluding hysterectomy. As participants progressed beyond menopause, the number of years since the final menstrual period were identified for analysis of the postmenopausal stage. Analyses have been conducted comparing early and later postmenopause as follows: <2 versus > =2 years [44]; 0–3 versus >3–11 years [45]; 0–5 versus 6–14 years [46].

Menopausal stage was identified at each assessment period, using the menstrual dates recorded at each study visit. This included the date of the current menstrual cycle (visits were conducted within 6 days of bleeding) and the dates for the 2 previous menstrual cycles, which were recorded at each visit. The 6 dates recorded at 2 visits provided consecutive menstrual dates for approximately 4 months in each assessment period. Additional confirmatory data were obtained from the daily symptoms diaries that participants recorded for one menstrual cycle at each assessment period (the diary date was used in cases of disagreement). Other confirmatory data included the reported number of menstrual periods between assessments, cycle length and the number of bleeding days, which were obtained in the structured interviews and the daily diaries.

Hormonal contraception, hysterectomy, pregnancy and breast feeding were exclusions at study enrollment. When these events occurred during the study, the information was coded and menopausal stage for these subjects was classified separately in categories of “hysterectomy”, “hormone use”, and “pregnancy/breast feeding” in the relevant assessment periods.

### Identification of final menstrual period (FMP)

The final menstrual period was identified retrospectively after 12 or more months of no menstrual bleeding. The FMP marked entry into the postmenopausal stage. The small number of participants with surgical menopause were categorized separately.

### Study adherence and attrition

Attrition occurred primarily in the early years of the study (Table 2). Nineteen percent (83/436) discontinued in the first 6 years. Half of these discontinued in the first year of the study, when they refused further participation or could not be located for the first follow-up evaluation. Only 10 % (42/436) discontinued in Phase 2 (years 7–10), and 4 % (18/436) discontinued in Phase 3 (years 11–14), for a total of 33 % attrition (143/436) over 14 years. Attrition through Period 14 was classified as lost to follow-up (*n* = 51), no reason given (*n* = 40), withdrew consent (*n* = 22), personal constraints or problems (*n* = 16), and deceased (*n* = 14).

**Table 1:** Outcome measures by study visits

Domain	Measure	Study visits^1^
Demographic	Interview questionnaire	1,3,5,7,9,11,13
Menstrual cycle:	Interview questionnaire	1,3,5,7,9,11,13
menstrual dates, cycle characteristics ovulation test		2,4,6,8,10,12
Menopausal symptoms	Symptom questionnaire^5^	all odd visits 1-27
	Daily symptom reports	all odd visits 1-27
Health: gynecologic, reproductive, medications, health practices/ behaviors	Interview questionnaire	all odd visits 1-27
General	SF-12 Health Survey^24^	all odd visits 1-27
Physical activity	Questionnaire^6^	4,12,16,20,22,24,25,27
	Kaiser Physical Activity Survey^37^	25,27
Sleep	SMH Sleep Questionnaire^8,9^	1,3,5,7,11,13,15,19
	WHI Insomnia Rating Scale^28^	19,21,23,25,27
	Apnea Prediction Index^29^	19,21,23,25,27
Mood and anxiety	PRIME-MD^15^	2,4,6,8,10,12
	Patient Health Questionnaire^16,17^	14,16,18,20,21,23,25 27
	CES-Depression Scale^18,19^	all odd visits 1-27
	Zung Anxiety Scale^20^	all odd visits 1-27
	Perceived Stress (PSS)^21^	2,4,6,8,10,12, 3–27 (odd visits)
Quality of Life	Q-les-Q^22^	all odd visits
	Utian QOL Scale^23^	21,25,27
Sexual function	Female Sexual Function Index^30^	15,17,19,22,24,26,28
	Interview Questionnaire:	
	Abuse	11,17,19,23
	Interest	17,19,21,23,25,27
	Libido	all odd visits 1-27
Vaginal	Urinary Tract Rating	
	(BFLUTS-SF)^38^	21,24,26,28
	Interview Questionnaire	
	Dryness	odd visits 3-27
	Urine leaks	odd visits 3-27
Social support	Interview Questionnaire	1, 9, 15, 19
	Social Adjustment Scale^31^	1,3,5,11,13,15,22
	Life Events	9,11,13,17,21,24,25,27
Cognition	Cognitive memory tests	all even visits 2-28
	Buschke-Fuld Selective	
	Reminding Test^32^	
	Digit Symbol Substitution^33^	
	Digit Symbol Copy^33^	
Diet	Dietary Assessment	
	Questionnaire^39^	4, 10, 12, 14, 18
	Interview Questionnaire	3,5,7,11,15,19-17 (odd)
Anthropomorphic	Measurement:	
	Height, weight, waist, hip	all odd visits 1-27
	Wrist	23,25,27
Hormones	Estradiol, follicle stimulating hormone, Luteinizing hormone, DHEAS, Testosterone, inhibin b	all visits 1-28
	Sex hormone binding globulin	all visits 13-28
	Anti-mullerian hormone	V1 to various endpoints
Genotypes		413 participants

**Table 2:** Study continuation and attrition by assessment period

PERIOD	CONTINUING, *N* (%)	DROPPED, *N* (%)
1	436	__
2	394 (90)	42 (10)
3-4	366 (84)	28 (6)
5-6	353 (84)	13 (3)
7-8	320 (73)	33 (8)
9-10	311 (71)	9 (2)
11-12	301 (69)	10 (2)
13-14	293 (67)	8 (2)
TOTAL	293 (67)	143 (33)

We conducted a systematic analysis of study participation in the first 4 years, as reported in Nelson et al. [47]. Nelson examined demographic, behavioral, psychosocial and hormonal variables of the study that included age, race (African-American and Caucasian), education, marital status, body mass index, depressive symptoms, menopausal symptoms, perceived stress, anxiety, and reproductive hormone levels. There were no racial differences in study participation. Reproductive hormone levels at baseline did not differ between active and dropout groups. Only 2 variables marginally differed between active participants and dropouts: the dropout group was less likely to have high school education and less likely to report menopausal symptoms. Attrition to date has not significantly differed by race.

### Effect of attrition on power

The pre-enrollment estimates for sample size indicated that a cohort of 300 women would detect clinically relevant differences in hormones and symptoms, using a 2-sided alpha error of 0.05 and 80 % power. We enrolled 436 women based on estimates of attrition in the first 4 years of the study. At the 12-year follow-up, 300 participants remained active and 293 participants remained active at Year 14.

We consider the home visits to be the key element in maintaining the high completion rate, which has far exceeded the original estimates. All data and blood samples were collected at home visits from enrollment through Period 14, resulting in minimal missing data throughout the study. Incentives were provided to the participants, such as a small payment and a gift (e.g., mugs, magnets, throws, etc.) at the completion of each visit. In the intervals between the annual follow-up assessments, study staff maintained contact with participants by sending birthday cards and holiday cards each year. Participants were closely monitored via mailings and telephone calls around the dates of estimated menses in order to schedule visits on days 2–6 of the menstrual cycle. If a participant could not be located by mail or telephone, an interviewer went to the most recent home address to make contact and/or to query neighbors for information to locate the participant.

### Data management

Two separate but related database management systems were established. The first Management Information System (MIS) addressed the internal study procedures. This included a database of all eligible women with the phone numbers, addresses, and persons who could be contacted on their behalf if the participant could not be located. The database also included a full register of the menstrual cycle dates reported by each participant at each visit and a record of the blood collections as they were stored in freezers or sent for assay. This database was used by the study coordinator to track and schedule the study visits, to identify the freezer location of blood samples, and to select and batch blood samples for laboratory assays.

The second MIS contained all interview and questionnaire data collected for the study. These data were coded as they were collected and entered into computer files. A computer technician performed range checks and other organizing procedures to prepare the data for statistical analysis.

### Potential bias and quality control

Menopausal symptom assessment is inherently based on subjective perception. In order to limit expectancy effects of menopause, the participants were told that the study was a women’s health study. Specific questions about menopause were embedded in questionnaires with many other health questions. The menopause symptom questionnaire was validated and administered within the much longer structured Interview Questionnaire that assessed many aspects of women’s health. We assessed the major symptoms of interest with *both* structured interview questions and more detailed validated self-report measures.

To reduce recall bias, the reference time frame for symptoms information was current, the data were collected concurrently with the blood draws, and participants additionally rated symptoms prospectively in daily symptom reports for one menstrual cycle (or month) at each assessment period.

To control for menstrual cycle effects in the hormone measures, all data were collected in the first 6 days of the menstrual cycle in cycling women. Neither the interviewers nor the participants had information from the study on the participants’ hormone levels, making it unlikely that the women reported their symptoms based on hormone information.

To assure systematic data collection, we used structured interviews, and the interviewers were trained in the use of standard probes. Review sessions were conducted with the interviewers on an ongoing basis as part of the quality control process to address problems, develop consistent probes and promote systematic data collection. All interviewers were trained for SCID interviews via taped SCID interviews and training sessions with an experienced SCID interviewer. Interviewers checked that data for completion at each home visit. In addition, the study coordinator further reviewed all collected data when the data were returned to the research site. In the event of missing data, interviewers re-contacted participants to complete the missing items.

### Representativeness of the cohort

The cohort participants were randomly identified by random digit dialing in a large metropolitan area of the U.S., with stratification to obtain equal numbers of African American and Caucasian women. The exclusion criteria limited the cohort to healthy women with a uterus and ovary at enrollment. Consequently the data are generalizable to generally healthy African American and Caucasian women in large urban areas of the U.S. who experience natural menopause. Findings may not be generalized to other racial groups or to women with surgical menopause, hormone users, serious illnesses or chronic disease without further studies.

## Discussion

The POAS cohort was established to address the limited scientific understanding of menopausal hormone changes and symptoms and their relationships to health and morbidity of mid-life women. The cohort followed the large epidemiologic Study of Women’s Health Across the Nation (SWAN), with specific aims to evaluate an earlier baseline of symptoms and hormones in late reproductive age women before they entered the transition to menopause. Annual evaluations followed the participants as they traversed the menopause transition. After 18 years of follow-up, the cohort remains viable with adequate statistical power for studies of the natural menopause transition and early postmenopausal years.

Strengths of the cohort addressed several limitations of earlier studies. The longitudinal data identified menopausal stages as they were observed rather than by long-term recall. Menopausal stages were based on the initial STRAW [42], utilizing bleeding patterns to classify premenopausal, transition and postmenopausal stages. Hormone levels and symptoms were measured concurrently. To control for menstrual cycle effects, all data, including the blood samples, were collected within days 2–6 of the menstrual cycle in cycling women. Menopausal stage was determined annually and, together with calendar age, can be analyzed to identify the independent effects of these key markers of menopausal status. Longitudinal assessments from a premenopausal baseline allowed *new-onset* symptoms to be identified in the menopause transition. Extensive symptom assessments included identifying psychiatric diagnoses to more clearly interpret menopausal symptoms. The cohort was population-based, participants were randomly-identified and in general good health for this study of behavioral and hormonal factors in relation to natural menopause.

Limitations to consider include the following: the cohort includes only Caucasian and African American women, who were enrolled in equal numbers, and does not include other racial or ethnic groups. Blood samples were collected in the early follicular phase of the menstrual cycle (days 2–6) to control for menstrual cycle effects, but do not include luteal phase measures and cannot describe across-cycle hormone effects. Hysterectomy and hormone use were exclusions at enrollment and in subsequent follow-up did not occur in sufficient numbers to analyze these factors. Attrition naturally occurred over the 18 years of follow up (described above), but the cohort remains viable with adequate statistical power.

More than 50 studies based on this cohort are published in medical journals at this time, demonstrating the relevance of these data to the clinical care of mid-life women. (See Additional file 1: Appendix 1). Several examples of the POAS data as they elucidate clinical issues are the following: studies of the timing and duration of hot flashes show that hot flashes can occur well before menopause and extend 10 or more years beyond menopause for sizeable numbers of women [48, 49]; data provide evidence for new-onset depressed mood in the menopause transition and show that the final menstrual period is pivotal in the premenopausal increase and postmenopausal decrease in depressive symptoms [44, 50, 51]; data show that poor sleep is common in the late reproductive years but suggest that only a small proportion of women experience increases in poor sleep in relation to the final menstrual period [28]; studies provide evidence of effects of obesity on reproductive hormones in the menopause transition [45, 52]; show the relationship between smoking and hot flashes as a function of genetic variation in sex-steroid metabolizing enzymes [46, 53]; indicate decreased libido in the menopause transition [54, 55]; menopause effects on verbal memory [56]; and anti-mullerian hormone as a predictor of time to menopause [57, 58].

In conclusion, longitudinal data are critical for the understanding of hormone changes and symptoms that are experienced by mid-life women and influence other physical conditions of aging. The POAS cohort and other recent cohorts of mid-life women exist to address questions about these menopausal changes. Increasing scientific information about the biological and behavioral changes associated with menopause contributes to improving the health care of women.

## Additional file

Additional file 1: **Appendix 1.** Publications of Data from the Penn Ovarian Aging Study (POAS). ( DOC 29.2 KB)

## Abbreviations

AMH: anti-mullerian hormone (AMH).
BMI: body mass index
DHEAS: dehydroepiandrosterone
FSH: follicle stimulating hormone
LH: luteinizing hormone
POAS: Penn Ovarian Aging Study
SHGB: sex hormone binding globulin
STRAW: Stages of Reproductive Aging Workshop
SWAN: Study of Women’s Health Across the Nation
T: testosterone
