# Supplementary material for: Methods in a longitudinal cohort study of late reproductive age women: the Penn Ovarian Aging Study (POAS)
Source: Womens Midlife Health. 2016 Jan 27;2:1. doi: 10.1186/s40695-016-0014-2 (PMC6299955; doi:10.1186/s40695-016-0014-2)
Supplement: Supplementary file 1 — Appendix 1. Publications of Data from the Penn Ovarian Aging Study (POAS). ( DOC 29.2 KB) [file 40695_2016_14_MOESM1_ESM.doc]

**APPENDIX 1**

**PUBLICATIONS OF DATA FROM THE PENN OVARIAN AGING STUDY (POAS)**

1**.** Grisso JA, Freeman EW, Maurin E, Garcia-Espana B, Berlin JA. Racial differences in menopause information and the experience of hot flashes. J Gen Intern Med. 1999 Feb;14(2):98-

103. PubMed PMID: 10051780.

2. Birken S, Santoro N, Maydelman Y, Kovalevskaya G, Lobo R, Freeman EW, Warren M, McMahon D, O'Connor J. Differences in urinary excretion patterns of the hLH beta core fragment in premenopausal, perimenopausal, and postmenopausal women. Menopause. 1999

Winter;6(4):290-8. PubMed PMID: 10614675.

3. Freeman EW, Grisso JA, Berlin J, Sammel M, Garcia-Espana B, Hollander L. Symptom reports from a cohort of African American and white women in the late reproductive years. Menopause. 2001 Jan-Feb;8(1):33-42. PubMed PMID: 11201513.

4. Manson JM, Sammel MD, Freeman EW, Grisso JA. Racial differences in sex hormone levels in women approaching the transition to menopause. Fertil Steril. 2001 Feb;75(2):297-304. PubMed PMID: 11172830.

5. Freeman EW, Sammel MD, Grisso JA, Battistini M, Garcia-Espagna B, Hollander L. Hot flashes in the late reproductive years: risk factors for Africa American and Caucasian women. J Womens Health Gend Based Med. 2001 Jan-Feb;10(1):67-76. PubMed PMID:

11224946.

6. Sammel MD, Wang Y, Ratcliffe SJ, Freeman E, Propert KJ. Models for within subject heterogeneity as predictors for disease. Prodeedings of the Annual Meeting of the American Statistical Association, August 5-9, 2001.

7. Battistini M, Freeman EW, Grisso JA, Sammel M, Hollander L, Garcia-Espana B. Pilot study of racial differences and longitudinal changes in inhibin B in the late reproductive years. Fertil Steril. 2002 Jan;77(1):193-5. PubMed PMID: 11779617.

8. Hollander LE, Freeman EW, Sammel MD, Berlin JA, Grisso JA, Battistini M. Sleep quality, estradiol levels, and behavioral factors in late reproductive age women. Obstet Gynecol. 2001 Sep;98(3):391-7. PubMed PMID: 11530118.

9. Morrison MF, Ten Have T, Freeman EW, Sammel MD, Grisso JA. DHEA-S levels and depressive symptoms in a cohort of African American and Caucasian women in the late reproductive years. Biol Psychiatry. 2001 Nov 1;50(9):705-11. PubMed PMID: 11704078.

10. Freeman EW, Sammel MD, Liu L, Martin P. Psychometric properties of a menopausal symptom list. Menopause. 2003 May-Jun;10(3):258-65. PubMed PMID: 12792299.

11. Sammel MD, Grisso JA, Freeman EW, Hollander L, Liu L, Liu S, Nelson DB, Battistini

M. Weight gain among women in the late reproductive years. Fam Pract. 2003

Aug;20(4):401-9. PubMed PMID: 12876110.

12. Freeman EW, Sammel MD, Liu L, Gracia CR, Nelson DB, Hollander L. Hormones and menopausal status as predictors of depression in women in transition to menopause. Arch

Gen Psychiatry. 2004 Jan;61(1):62-70. PubMed PMID: 14706945.

13. Gracia CR, Sammel MD, Freeman EW, Liu L, Hollander L, Nelson DB. Predictors of decreased libido in women during the late reproductive years. Menopause. 2004 Mar- Apr;11(2):144-50. PubMed PMID: 15021443.

14. Nelson DB, Sammel MD, Freeman EW, Liu L, Langan E, Gracia CR. Predicting participation in prospective studies of ovarian aging. Menopause. 2004 Sep-Oct;11(5):543-8. PubMed PMID: 15356407.

15. Freeman EW, Sammel MD, Rinaudo PJ, Sheng L. Premenstrual syndrome as a predictor of menopausal symptoms. Obstet Gynecol. 2004 May;103(5 Pt 1):960-6. PubMed PMID: 15121571.

16. Gracia CR, Freeman EW. Acute consequences of the menopausal transition: the rise of common menopausal symptoms. Endocrinol Metab Clin North Am. 2004 Dec;33(4):675-89. Review. PubMed PMID: 15501640.

17. Freeman EW, Sammel MD, Gracia CR, Kapoor S, Lin H, Liu L, Nelson DB. Follicular phase hormone levels and menstrual bleeding status in the approach to menopause. Fertil Steril. 2005 Feb;83(2):383-92. PubMed PMID: 15705379.

18. Gracia CR, Sammel MD, Freeman EW, Lin H, Langan E, Kapoor S, Nelson DB. Defining menopause status: creation of a new definition to identify the early changes of the menopausal transition. Menopause. 2005 Mar;12(2):128-35. PubMed PMID: 15772558.

19. Freeman EW, Sammel MD, Lin H, Gracia CR, Kapoor S, Ferdousi T. The role of anxiety and hormonal changes in menopausal hot flashes. Menopause. 2005 May- Jun;12(3):258-66. PubMed PMID: 15879914.

20. Nelson DB, Sammel MD, Freeman EW, Gracia CR, Liu L, Langan E. Tubal ligation does not affect hormonal changes during the early menopausal transition. Contraception.

2005 Feb;71(2):104-10. PubMed PMID: 15707559.

21. Gracia CR, Freeman EW, Sammel MD, Lin H, Nelson DB. The relationship between obesity and race on inhibin B during the menopause transition. Menopause. 2005 Sep- Oct;12(5):559-66. Epub 2005 Sep 1. PubMed PMID: 16145310.

22. Freeman EW, Sammel MD, Lin H, Nelson DB. Associations of hormones and menopausal status with depressed mood in women with no history of depression. Arch Gen Psychiatry. 2006 Apr;63(4):375-82. PubMed PMID: 16585466.

23. Freeman EW, Gracia CR, Sammel MD, Lin H, Lim LC, Strauss JF 3rd. Association of anti-mullerian hormone levels with obesity in late reproductive-age women. Fertil Steril.

2007 Jan;87(1):101-6. Epub 2006 Nov 15. PubMed PMID: 17109858.

24. Gracia CR, Freeman EW, Sammel MD, Lin H, Mogul M. Hormones and sexuality during transition to menopause. Obstet Gynecol. 2007 Apr;109(4):831-40. PubMed PMID:

17400843.

25. Freeman EW, Sammel MD, Lin H, Gracia CR, Pien GW, Nelson DB, Sheng L. Symptoms associated with menopausal transition and reproductive hormones in midlife women. Obstet Gynecol. 2007 Aug;110(2 Pt 1):230-40. PubMed PMID: 17666595.

26. Schmitz KH, Lin H, Sammel MD, Gracia CR, Nelson DB, Kapoor S, DeBlasis TL, Freeman EW. Association of physical activity with reproductive hormones: the Penn Ovarian Aging Study. Cancer Epidemiol Biomarkers Prev. 2007 Oct;16(10):2042-7. Epub 2007 Sep

28. PubMed PMID: 17905944.

27. Freeman EW, Sammel MD, Lin H, Gracia CR, Kapoor S. Symptoms in the menopausal transition: hormone and behavioral correlates. Obstet Gynecol. 2008 Jan;111(1):127-36. PubMed PMID: 18165401.

28. Nelson DB, Sammel MD, Freeman EW, Lin H, Gracia CR, Schmitz KH. Effect of physical activity on menopausal symptoms among urban women. Med Sci Sports Exerc.

2008 Jan;40(1):50-8. PubMed PMID: 18091021.

29. Su HI, Sammel MD, Freeman EW, Lin H, DeBlasis T, Gracia CR. Body size affects measures of ovarian reserve in late reproductive age women. Menopause. 2008 Sep- Oct;15(5):857-61. PubMed PMID: 18427357; PubMed Central PMCID: PMC2821936.

30. Pien GW, Sammel MD, Freeman EW, Lin H, DeBlasis TL. Predictors of sleep quality in women in the menopausal transition. Sleep. 2008 Jul 1;31(7):991-9. PubMed PMID:

18652094; PubMed Central PMCID: PMC2491505.

31. Gopal M, Sammel MD, Pien G, Gracia C, Freeman EW, Lin H, Arya L. Investigating the associations between nocturia and sleep disorders in perimenopausal women. J Urol.

2008 Nov;180(5):2063-7. Epub 2008 Sep 19. PubMed PMID: 18804245; PubMed Central

PMCID: PMC2824249.

32. Gopal M, Sammel MD, Arya LA, Freeman EW, Lin H, Gracia C. Association of change in estradiol to lower urinary tract symptoms during the menopausal transition. Obstet Gynecol. 2008 Nov;112(5):1045-52. PubMed PMID: 18978104; PubMed Central PMCID: PMC2813704.

33. Sammel MD, Freeman EW, Liu Z, Lin H, Guo W. Factors that influence entry into stages of the menopausal transition. Menopause. 2009 Nov-Dec;16(6):1218-27. PubMed PMID: 19512950; PubMed Central PMCID: PMC2783664.

34. Su HI, Freeman EW. Hormone changes associated with the menopausal transition. Minerva Ginecol. 2009 Dec;61(6):483-9. Review. PubMed PMID: 19942836.

35. Su HI, Sammel MD, Green J, Velders L, Stankiewicz C, Matro J, Freeman EW, Gracia CR, DeMichele A. Antimullerian hormone and inhibin B are hormone measures of ovarian function in late reproductive-aged breast cancer survivors. Cancer. 2010 Feb 1;116(3):592-9. PubMed PMID: 19918920; PubMed Central PMCID: PMC2815049.

36. Freeman EW, Sammel MD, Lin H, Gracia CR. Obesity and reproductive hormone levels in the transition to menopause. Menopause. 2010 Jul;17(4):718-26. PubMed PMID:

20216473; PubMed Central PMCID: PMC2888623.

37. Su HI, Sammel MD, Springer E, Freeman EW, Demichele A, Mao JJ. Weight gain is associated with increased risk of hot flashes in breast cancer survivors on aromatase

inhibitors. Cancer Res Treat. 2010 Nov;124(1):205-11. Epub 2010 Feb 25. PubMed PMID:

20182796; PubMedCentral PMCID: PMC3670946.

38. Rebbeck TR, Su HI, Sammel MD, Lin H, Tran TV, Gracia CR, **Freeman EW**. Effect

of hormone metabolism genotypes on steroid hormone levels and menopausal symptoms in a prospective population-based cohort of women experiencing the menopausal transition. Menopause. 2010 Sep-Oct;17(5):1026-34. PubMed PMID: 205058012PubMed Central PMCID: PMC3072891.

39. Nelson DB, Sammel MD, Patterson F, Lin H, Gracia CR, Freeman EW. Effects of reproductive history on symptoms of menopause: a brief report. Menopause. 2011; 18 (10):

1143 – 8. Epub 2011 May 4. PubMed PMID: 21552165; PubMed Central PMCID: PMC3158958.

40. Freeman EW, Sammel MD, Lin H, Liu Z, Gracia CR. Duration of menopausal hot flushes and associated risk factors. Obstet Gynecol. 2011 May;117(5):1095-104. PubMed PMID: 21508748; PubMed Central PMCID: PMC3085137.

41. Morrison MF, Freeman EW, Lin H, Sammel MD. Higher DHEA-S (dehydroepiandrosterone sulfate) levels are associated with depressive symptoms during the menopausal transition: results from the PENN Ovarian Aging Study. Arch Womens Ment Health. 2011 Oct;14(5):375-82. doi: 10.1007/s00737-011-0231-5. Epub 2011 Jul 20. PubMed PMID: 21773816; PubMed Central PMCID: PMC3690802.

42. **Freeman EW**, Sammel MD, Lin H. Gracia CR. Anti-mullerian hormone as a predictor of time to menopause in late reproductive age women. J Clin Endocrinol Metab. 2012; 97 (5): 1673-80. Epub 2012 Feb 29. PMID 22378815; PubMed Central PMCID: PMC3339896.

43. Butts SF, Freeman EW, Sammel MD, Queen K, Lin H, Rebbeck TR. Joint effects of smoking and gene variants involved in sex steroid metabolism on hot flashes in late reproductive-age women. J Clin Endocrinol Metab 2012; 97 (6): 1032-42. Epub 2012

Mar 30. PMID 22466345; PubMedCentral PMCID: PMC3387409.

44. Freeman EW, Sammel MD, Lin H, Boorman DW, Gracia CR. Contribution of the rate of change of antimüllerian hormone in estimating time to menopause for late reproductive- age women. Fertil Steril 2012; 98 (5):1254-9.e1-2. Epub 2012 Aug 24. PMID: 22921911

PubMed Central PMCID: PMC3478472.

45. Epperson CN, Sammel MD, **Freeman EW**. Menopause effects on verbal memory: findings from a longitudinal community cohort. J Clin Endocrinol Metab 2013; 58(9): 3829-3838. Epub 2013

Jul 8. PubMed PMID: 23836935. PubMed Central PMCID: PMC3763981.

46. Senapati S, Gracia CR, **Freeman EW**, Sammel MD, Lin H, Kim C, Schwab RJ, Pien GW. Hormone variations associated with quantitative fat measures in the menopausal transition. Climacteric 2014; 17 (2):183-190. Epub 2013 Oct 28. PMID: 24066661.

47. Freeman EW, Sammel MD, Boorman DW. The longitudinal pattern of depressive symptoms around natural menopause. JAMA Psychiatry 2014; 71 (1): 36-43. Epub: 2013 Nov 13. PMID:

24227182.

48. Butts SF, Sammel MD, Greer C, Rebbeck TR, Boorman D, Freeman EW. Cigarettes, genetic background and menopausal timing: the presence of single nucleotide polymorphisms in Cytodhrome

P450 genes hastens the onset of natural menopause in European American smokers. Menopause

2014; 21 (7):694-701. Epub 2014 Jan 20. PMID: 24448104.

49. Freeman EW, Sammel MD, Sanders RJ. Risk of long term hot flashes after natural menopause: evidence from the Penn Ovarian Aging Study. Menopause 2014; 21 (9): 924-932. Epub 2014 Jan 28. PMID: 24473530.

50. Freeman EW, Sammel MD, Gross SA, Pien GW. Poor sleep in relation to natural menopause: a population-based 14-year follow-up of midlife women. Menopause 2015; 22 (7): 719-726. Epub

2014 Dec 29. PMID: 25549066. PubMed Central PMCID: PMC4481144.

51. Milman LW, Sammel MD, Barnhart KT, Freeman EW, Dokras A. Higher serum total testosterone levels correlate with increased risk of depressive symptom ms in Caucasian women through the entire menopausal transition. Psychoneuroendocrinology 2015; 62: 107-13. PMID:

26280374.

52. Jiang B, Elliott MR, Sammel MD, Wang NA. Joint modeling of cross-sectional health outcomes and longitudinal predictors via mixtures of means and variances. Biometrics 2015; 71(2):

487-97.

53. Jiang B, Wang N, Sammel MD, Elliott MR. Modeling short- and long-term characteristics of follicle stimulating hormone as predictors of severe hot flashes in the Penn Ovarian Aging Study. Applied Statistics 2015; in press.
